# Blocking IL-33 decelerates cartilage degeneration in knee osteoarthritis through mice model

**DOI:** 10.1371/journal.pone.0301199

**Published:** 2024-08-22

**Authors:** Fan Wu, Siyuan Zhang, Rujie Zhuang, Chuanxiao Hu, Kangxiang Zhu

**Affiliations:** 1 Department of Orthopaedics, Quzhou Traditional Chinese Medicine Hospital at the Junction of Four Provinces Affiliated to Zhejiang Chinese Medical University, Quzhou, Zhejiang, China; 2 Department of Orthopaedics, The First Affiliated Hospital of Zhejiang Chinese Medical University (Zhejiang Provincial Hospital of Traditional Chinese Medicine), Hangzhou, Zhejiang, China; 3 Department of Neuroelectrophysiology, People’s Hospital of Quzhou, Quzhou, Zhejiang, China; Yarmouk University, JORDAN

## Abstract

**Introduction:**

Osteoarthritis (OA) is a chronic inflammatory disease where pro-inflammatory cytokines, damage-associated molecular patterns and macrophages play a crucial role. However, the interaction of these mediators, the exact cause, and the treatment of knee osteoarthritis (KOA) are still unclear. Moreover, the interaction of interleukin (IL)-33, platelet-derived growth factor-BB (PDGF-BB), and matrix metalloproteinase-9 (MMP-9) with other factors in the pathogenesis of KOA has not been elaborately explored.

**Method:**

Therefore, in this study, we analyzed the expression of IL-33, PDGF-BB, and MMP-9 in the knee cartilage tissue of model mice, murine KOA was induced by using the destabilization of the medial meniscus (DMM) model.

**Results:**

Compared with the sham operation control group, the expression levels of PDGF-BB, IL-33, and MMP-9 were increased significantly, and the pathological sections showed obvious cartilage damage. Additionally, we assessed the levels of IL-33 and MMP-9 expression in the knee joint of KOA model mice following intervention with PDGF-BB antibody, and we found that the expression level of MMP-9 was reduced following intervention with IL-33 antibody. When the effects of the three antibodies were compared in a mouse disease model, it was discovered that the IL-33 antibody could dramatically lower the relative expression level of MMP-9, resulting in the least amount of cartilage damage and improved protection. In conclusion, inhibiting IL-33 can significantly lower inflammatory factor levels in the knee joint, including IL-33 and MMP-9, and it can improve cartilage breakdown in osteoarthritis of the knee.

**Conclusion:**

Overall, the results indicate that IL-33 has a therapeutic function in the treatment of knee osteoarthritis and may be a novel target for treatment of the underlying causes of KOA. Additionally, PDGF-BB might be an upstream pathway of IL-33, and KOA’s MMP-9 is an downstream pathway of IL-33.

## Introduction

Osteoarthritis (OA) stands out as one of the most prevalent degenerative joint diseases, typically afflicting older adults who experience characteristic clinical symptoms, including joint pain, swelling, stiffness, and reduced mobility [1]. The global prevalence of OA exceeds 303 million individuals, as indicated by a 2017 statistical study [2]. This statistic underscores the significant impact of OA on patients’ quality of life and its substantial financial burden on individuals, families, and communities. As the world’s population ages and life expectancy increases, OA poses a substantial threat to public health and society [3,4].

Although the precise cause of OA remains elusive, several risk factors, particularly in the context of knee osteoarthritis (KOA), contribute to its development. These factors encompass traumatic injury, aging, obesity, genetic predisposition, abnormal mechanical stress, and inflammation stemming from infection or surgery [5]. Recent research has illuminated the multifaceted impact of KOA on various joint structures, including the synovium, articular cartilage, subchondral bone, intra-articular fat pads, and the overall joint architecture [6]. Of particular significance are the fibrocartilage structures that provide intra-articular support, as well as the chronic inflammation of these structures within the knee joint [7,8]. Progressive destruction of articular cartilage and subchondral sclerosis are key pathological hallmarks of KOA, signifying irreversible articular cartilage degradation [9].

Current research on KOA predominantly revolves around the inflammatory response, which is often incited by immune cells with an inflammatory profile. Cytokines play a central role in the pathophysiological processes leading to the onset and progression of KOA [10,11]. Notable examples include proinflammatory cytokines such as tumor necrosis factor-alpha (TNF-α), interleukin-1 beta (IL-1β), and interleukin-6 (IL-6), produced by immune cells and macrophages, as well as anti-inflammatory cytokines like interleukin-4 (IL-4), interleukin-10 (IL-10), and interleukin-37 (IL-37) [12–15]. These cytokines collectively contribute to the pathogenesis of arthritis. Interleukin-33 (IL-33), a member of the IL-1 cytokine family [16], is a pivotal player in cellular interactions within the inflammatory microenvironment, found in conditions such as tumors, respiratory, and digestive systems [13,17,18]. Recent studies have linked IL-33 to KOA progression, as it mediates the production and secretion of matrix metalloproteinases (MMPs), particularly MMP-9 and MMP-13, by chondrocytes via the ST2 receptor [19]. This process has direct or indirect effects on articular cartilage, leading to abnormal cell metabolism and secretion and disrupting the homeostasis of the internal articular cartilage environment [20–23]. Nonetheless, further research is required to fully understand the activation and mechanism of IL-33. It is postulated that abnormal subchondral bone angiogenesis, involving the formation of new blood vessels from preexisting ones in osteoarthritic joints, promotes KOA development and serves as an early manifestation of the disease, occurring before articular cartilage damage [24,25]. One proposed theory suggests that increased secretion of platelet-derived growth factor-BB (PDGF-BB) by subchondral mononuclear osteoclasts activates platelet-derived growth factor receptor-β (PDGFR-β) signaling in pericytes, leading to neovascularization and hastening cartilage degeneration [26,27]. Additionally, it has been observed that PDGF-BB can stimulate pericytes to release IL-33 through PDGFR-β in other diseases [28]. Nevertheless, the involvement of IL-33 in KOA and the specific pathways involved require further investigation, as it remains uncertain whether PDGF-BB can induce an increase in IL-33 through this pathway during the progression of KOA.

In summary, the primary objective of this study is to employ animal experiments to unravel the mechanisms and intrinsic connections between IL-33, PDGF-BB, and MMP-9 in KOA. Furthermore, we aim to assess the therapeutic effects of various antibodies on KOA in mice, with the ultimate goal of providing insights that can guide new clinical target therapies.

## Materials and methods design

### Ethical statement

The study protocol and the utilization of animals in this research were formally approved by the Institutional Animal Care and Use Committee (IACUC) of Zhejiang Chinese Medicine University and conducted in strict adherence to the committee’s guidelines (Approval No: IACUC-20210201-05). The ethical treatment of the animals was paramount in this study.

All experimental animals were housed in controlled laboratory environments, ensuring their well-being. They were accommodated in individually ventilated cages and provided with standard laboratory conditions, including ad libitum access to both food and water. The room temperature was carefully maintained within a range of 18–22°C to ensure their comfort and minimize any stress-related effects.

A total of eighty-four healthy C57BL/6 mice, each of them aged 8 weeks and weighing within the range of 25±5 grams, were thoughtfully selected for this study. These mice were procured from the reputable Animal Center of Zhejiang Chinese Medical University, operating under a valid production license (License No: SCXK 2021–0003) and meeting the necessary criteria for animal experimentation conditions (Qualification Certificate No: SYXK 2021–0012).

### Reagents and materials

We sourced essential reagents and materials from reputable suppliers. The TRIzol® Plus RNA Purification Kit and SuperScript™ III First-Strand Synthesis Super Mix for qRT-PCR were obtained from Invitrogen (USA). Applied Biosystems (USA) provided the Power SYBR® Green PCR Master Mix. The MMP-9 antibody reagent was supplied by Gene Operation (USA), while the PDGF-BB antibody, IL-33 antibody, and T-PER Tissue Protein Extraction Reagent were acquired from Thermo Fisher (Shanghai, China). The Transfer Film and BCA Protein Assay Kit were procured from Millipore Corporation (USA) and Beyotime (China), respectively. Additionally, the Laboratory Animal Research Center of Zhejiang Chinese Medical University provided the remaining necessary testing equipment.

### Establishment of animal models and grouping

We established an experimental knee osteoarthritis (KOA) model in rats by utilizing the destabilized medial meniscus (DMM) induction method [29,30]. Eighteen male C57BL/6 mice, each 8 weeks old and weighing 25 grams, were randomly assigned to two groups: the surgical model group (DMM group) and the surgical control group (Sham group).

In the DMM group, we surgically severed the medial meniscus ligament of the right knee joint in the mice, preserving the patellar ligament. Prior to the procedure, we administered 0.3 ml of sodium pentobarbital solution (0.15 mg/ml) intraperitoneally to ensure full anesthesia. After the surgery, we injected 800,000 units of penicillin into the gluteus maximus. The KOA model in these mice was established after four weeks, adhering to the principles of minimizing pain and distress in animals. Euthanasia was performed using a 5% isoflurane inhalation for more than one minute. Absence of heartbeat and breathing were the criteria used to confirm death. The deceased mice were then transferred to the designated disposal area at the Laboratory Animal Center of Zhejiang University of Traditional Chinese Medicine. In the sham-operated control group, the integrity of the ligament and meniscus tissues was preserved using an identical surgical technique. A medial incision was made in the right knee joint of the mice, but without cutting the patellar ligament or the medial meniscus ligament. The medial capsule and skin were sutured layer by layer, and an injection of 800,000 units of penicillin was administered in the gluteus maximus.

In a separate series of experiments, we randomly divided 8-week-old male C57BL/6 mice, weighing 25±5 grams, into five groups: MMP-9 antibody group, PDGF-BB antibody group, IL-33 antibody group, blank group, and model control group, with 12 mice in each group. The blank group underwent a sham operation, while the other four groups of mice underwent KOA surgery to establish the model. The surgical procedure was performed as previously described.

### Administration of antibody reagents to animals

Each antibody group received injections of 2 μg of antibody reagent, which were dissolved in a 20 μl phosphate buffer saline (PBS) solution, into the right knee joint cavity of the mice once a week for four consecutive weeks. The control group, over the same period, received injections of PBS into the right knee joint cavity. It’s important to note that the control group underwent a simulated procedure but did not receive any therapeutic intervention.

### Tissue processing and staining

The right knee tissues from euthanized mice were initially treated with a neutral buffered formaldehyde solution (pH 7.4) for 24 hours, followed by a 30-minute rinse with distilled water. To decalcify, we subjected the samples to Ethylene Diamine Tetraacetic Acid (EDTA) solution at room temperature once a week for four weeks. Subsequently, the samples were dehydrated over a period of 4–6 weeks, with a 30-minute PBS rinse and immersion in a gradient of ethanol solutions (100%, 95%, and 75%) for 3 minutes each.

Special paraffin used for preparing pathological sections was melted at 58–60°C for 1 hour, three times in total. The resultant samples were cut into sections approximately 3–4 μm thick, followed by baking in an oven (60–62°C; model DHG-9140A) for 2 hours. Next, the sections underwent two dewaxing steps in xylene, each lasting 3 minutes, followed by washing with anhydrous ethanol for 3 minutes.

The sections were then immersed sequentially in 90%, 80%, and 70% ethanol for 1–3 minutes, followed by a water rinse for 1 minute. Subsequently, the sections were stained with hematoxylin for 10 minutes at room temperature, followed by a 2-minute rinse with distilled water. This was followed by staining with eosin for 1 minute at room temperature.

Finally, we immersed the samples in an ethanol gradient (80%, 95%, and 100%) for 3 minutes each and performed two rounds of treatment with xylene for 5 minutes each. The sections were sealed using neutral gum. Observation of the cartilage structure and chondrocytes was conducted under a microscope with field of view settings at 50x and 100x.

### Western blotting

The knee joint tissues were treated with RIPA lysis buffer (Thermo, Shanghai, China) containing 1% phosphatase inhibitor cocktail and 1% protease inhibitor cocktail for 30 minutes. Following centrifugation at 12,000 x g in a 4°C refrigerated centrifuge for 10 minutes, the supernatants were collected. The total protein was quantified using a BCA Protein Assay Kit (Thermo, Shanghai, China).

The protein samples were electrophoretically separated through sodium dodecyl sulfate-polyacrylamide gel electrophoresis (SDS-PAGE) and subsequently transferred to polyvinylidene difluoride (PVDF) membranes. The PVDF membrane was then blocked in TBS-T (containing 5% BSA) for 1 hour at room temperature.

After blocking, the membrane underwent three washes with phosphate buffer solution (PBS) for antibody incubation. The different primary antibodies were applied and incubated at 4°C overnight. The primary antibodies used were as follows: anti-IL-33 (cat. no. ab187060; 1:1,000; Abcam), anti-PDGF-BB (cat. no. ab178409; 1:2,000; Abcam), anti-MMP-9 (cat. no. ab228402; 1:1,000; Abcam), anti-MMP-13 (cat. no. ab84594; 1:1,000; Abcam), and anti-GAPDH (cat. no. ab181602; 1:10,000; Abcam).

The membrane was subsequently washed with PBS-T and peroxidase-labeled anti-Mouse IgG (cat. no. 31160; 1:5,000; Thermo Pierce) or anti-Rabbit IgG (cat. no. 31210; 1:5,000; Thermo Pierce) was added and shaken for 1 hour. Following three additional PBS-T washes, each lasting 10 minutes, proteins were detected using chemiluminescence (ECL) reagent (Thermo, Shanghai, China).The intensities of protein bands in the Western blot were quantified using ImageJ software (version 1.46, National Institutes of Health).

### Real-Time Quantitative PCR (qRT-PCR)

RNA was extracted from mouse knee tissue cell samples following the manufacturer’s protocol using the TRIzol technique. To synthesize cDNA, the SuperScript kit was employed, and the resulting cDNA was utilized for real-time quantitative PCR analysis. The synthesis of cDNA was carried out by Sangon Biotech (Shanghai, China) using Primer Premier 6.0 and Beacon Designer 7.8 detection systems. The primer sequences can be found in Table 1. SYBR Green PCR Master Mix was employed for the detection process. Each sample was tested in triplicate, and the relative expression level of each gene factor was calculated using the 2^-ΔΔCT method.

**Table 1:** Genes and primer sequences for real-time PCR detection.

Gene	Genbank Accession	Primer Sequences(5’to3’)	Size(bp)	Annealing(°C)
Mouse GAPDH	GU214026.1	GAAGGTCGGTGTGAACGGATTTG CATGTAGACCATGTAGTTGAGGTCA	127	60
Mouse MMP-9	NM_013599.4	GGGGTTTCTGTCCAGACCAAG CTGGATGCCGTCTATGTCGTCT CCCTGAGTACATACAATGACCAATC	167	60
Mouse IL-33	NM_001164724.2	GAGACTCATAGTAGCGTAGTAGCA CCACTCCATCCGCTCCTTT	126	60
Mouse PDGF-BB	NM_011057.4	CCCTCGAGATGAGCTTTCCAA	133	60

### Statistical methods

For group comparisons, an independent sample t-test was employed, with all data represented as mean ± standard deviation (MEAN±SD). A one-way analysis of variance (ANOVA) was used for comparisons between multiple groups. Pairwise comparisons between multiple groups were performed using the LSD and S-N-K methods. A significance level of *P*<0.05 indicated a statistically significant difference, while *P*<0.01 denoted a highly significant statistical difference.

## Results

### Elevated levels of IL-33, PDGF-BB, and MMP-9 in murine KOA

#### Impact on cartilage degradation in murine KOA

**Fig 1:**
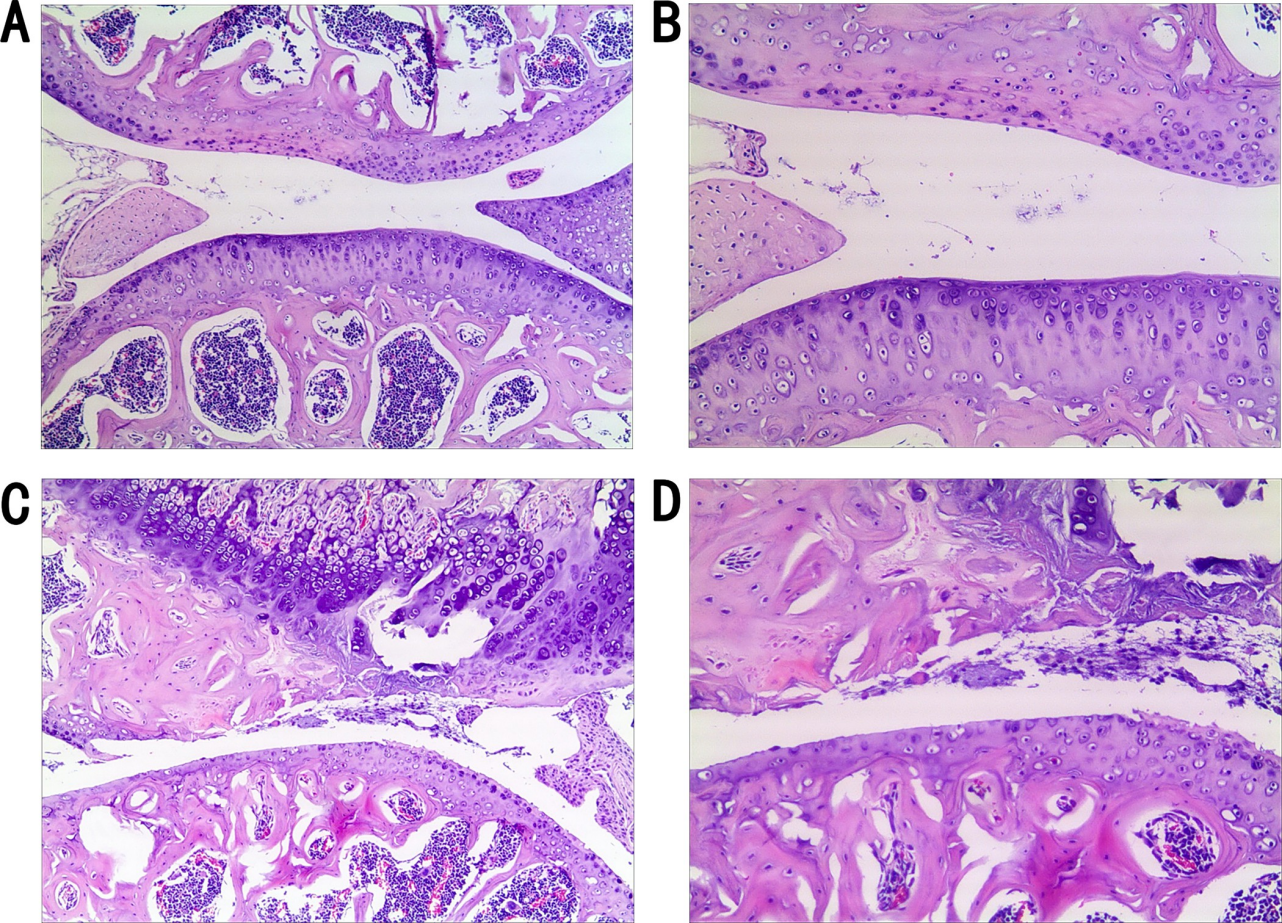
Comparative pathological analysis of murine knee cartilage: Control vs. Surgical modeling group. Comparison of pathological section of mouse knee cartilage between experimental and control groups. Murine KOA right knee cartilage pathological sections in the control group (A, B), and the surgical modeling group (C, D) (both 50x and 100x visual field comparison).

Examination of the pathological sections from the right knee joints of mice in the surgical model group, four weeks post-modeling, revealed severe damage to the cartilage structure. The surface appeared rough, chondrocytes were irregularly arranged, some chondrocytes had undergone apoptosis, and there were evident cavities. The bone trabeculae were also found to be incomplete (Fig 1A and 1B). In contrast, the pathological sections from the control group, which underwent a sham operation four weeks prior, showed a largely intact and smooth cartilage structure. There was no chondrocyte necrosis or damage, and the trabecular architecture remained intact (Fig 1C and 1D).

#### Relative expression levels of inflammatory factors in murine KOA

**Fig 2:**
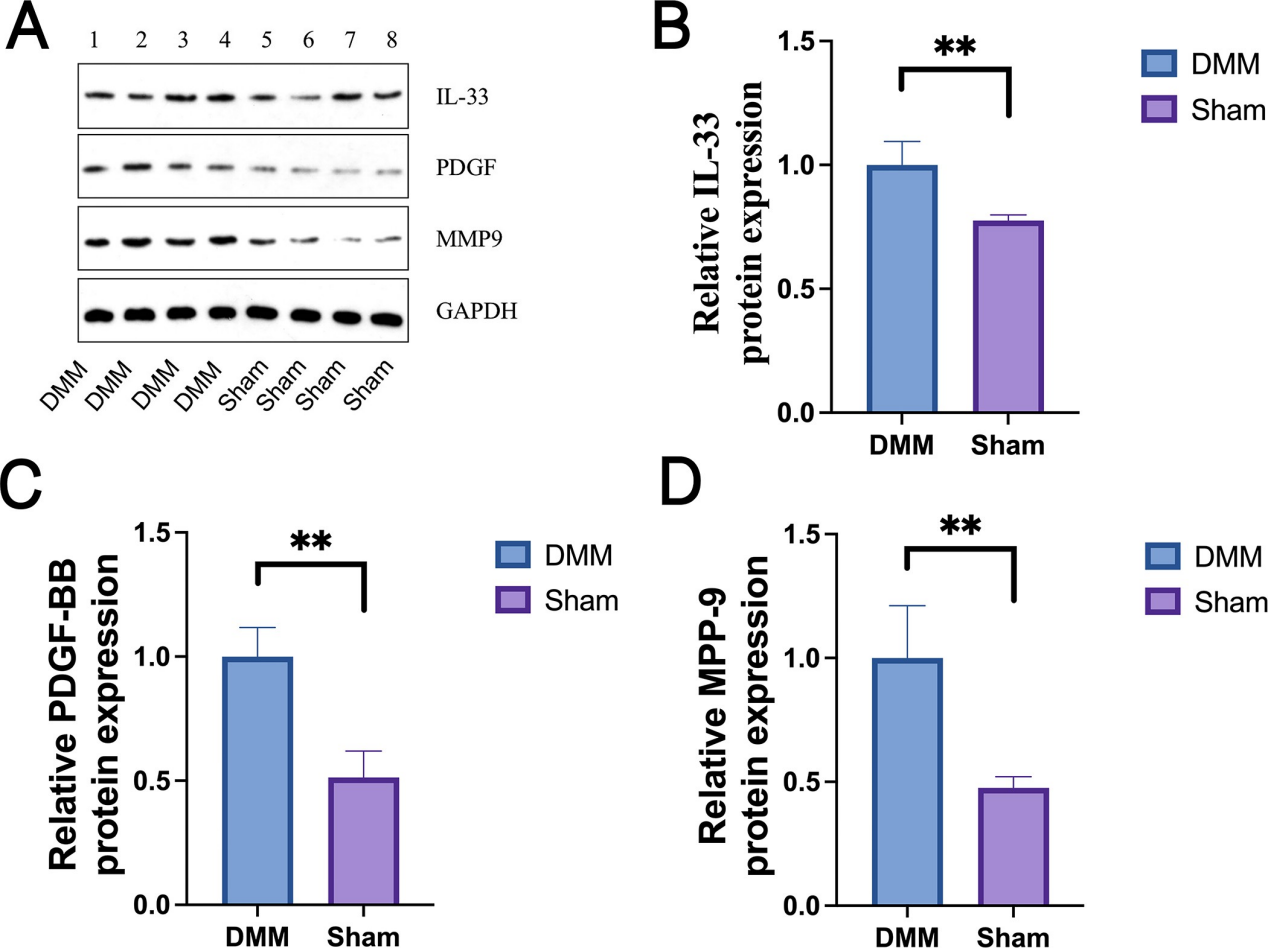
Differential expression of PDGF-BB, IL-33, and MMP-9 in mouse knee cartilage: Western blot and qRT-PCR analysis. In the mouse knee cartilage, PDGF-BB, IL-33, and MMP-9 are expressed. (A) A Western blot study of the mouse knee joint’s IL-33, PDGF-BB, and MMP-9 of protein levels. (Table 2) The ratio of the control group (Sham) and the inflammatory factor mRNA expression in mice knee samples was significantly different (P<0.01) using quantitative real-time polymerase chain reaction analysis of PDGF-BB (C), IL-33 (B), and MMP-9 (D). Independent sample t test was used to evaluate the data, and ** denotes incredibly significant differences (P<0.01).

Real-time PCR and western blot analysis were used to determine the relative levels of expression of inflammatory factors in murine KOA. There was a significant increase in protein levels of IL-33, PDGF-BB, and MMP-9 in murine KOA (Fig 2A). A comparison using an independent sample t-test revealed that the relative expression levels of PDGF-BB, IL-33, and MMP-9 in the surgical model group (DMM) were significantly elevated (Table 2). The DMM group exhibited significantly higher mRNA expression levels of PDGF-BB, IL-33, and MMP-9 compared to the control group (Sham) (P<0.01, Fig 2B–2D).

**Table 2:** Relative mRNA expression levels (Mean ± SD).

Grouping	The control group (Sham)	Model group (DMM)
Cases	9	9
PDGF-BB	55.92540 ± 10.27673	248.56220 ± 32.22060 * *
IL-33	26.24670 ± 4.10570	72.51440 ± 13.31255 * *
MMP-9	0.67440 ± 0.24815	6.29000 ± 1.16181 * *

### Reduction of inflammation and cartilage degradation with specific antibody treatment

#### Preservation of cartilage in murine KOA

**Fig 3:**
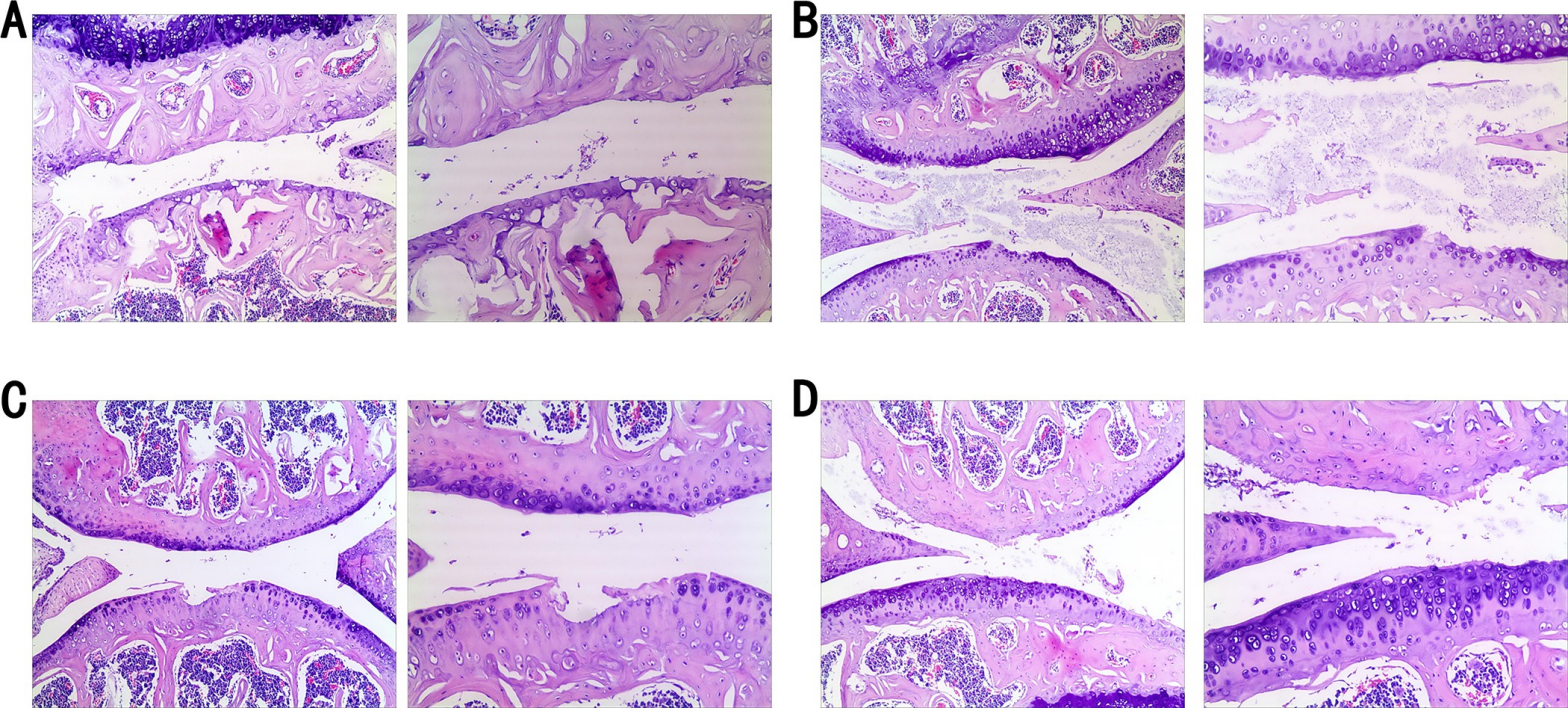
Effects of antibody intervention on pathological changes in mouse knee cartilage: MMP-9, PDGF-BB, and IL-33 analysis. Observations on pathological sections of mouse knee cartilage after antibody intervention. The pathological sections of the cartilage in the control group (A), the pathological sections in the mmp-9 antibody group (B), the PDGF-BB antibody group (C), and the IL-33 antibody group (D), (both 50x and 100x visual field comparison).

Four weeks after sham surgery, the cartilage in the control group remained largely intact, with a smooth surface and no necrosis or harm to chondrocytes. In contrast, the cartilage in the control group, after four weeks of modeling, showed structural degradation, an uneven surface, disorganized chondrocytes, increased chondrocyte mortality, and cavities (Fig 3A).

Pathological sections from the MMP-9 antibody group, after four weeks of therapy, displayed some cartilage damage, but the surface remained relatively smooth, with only a few apoptotic chondrocytes (Fig 3B). The PDGF-BB antibody group’s pathological sections, four weeks after injection, showed unevenly dispersed chondrocytes, with a minor proportion undergoing apoptosis (Fig 3C). The cartilage structure was also affected. Finally, the IL-33 antibody group’s pathological sections, four weeks into therapy, revealed a relatively smooth cartilage surface, fewer apoptotic chondrocytes, and mostly unaffected cartilage structure (Fig 3D).

#### Decreased expression of inflammatory factors after four weeks of antibody treatment

**Fig 4:**
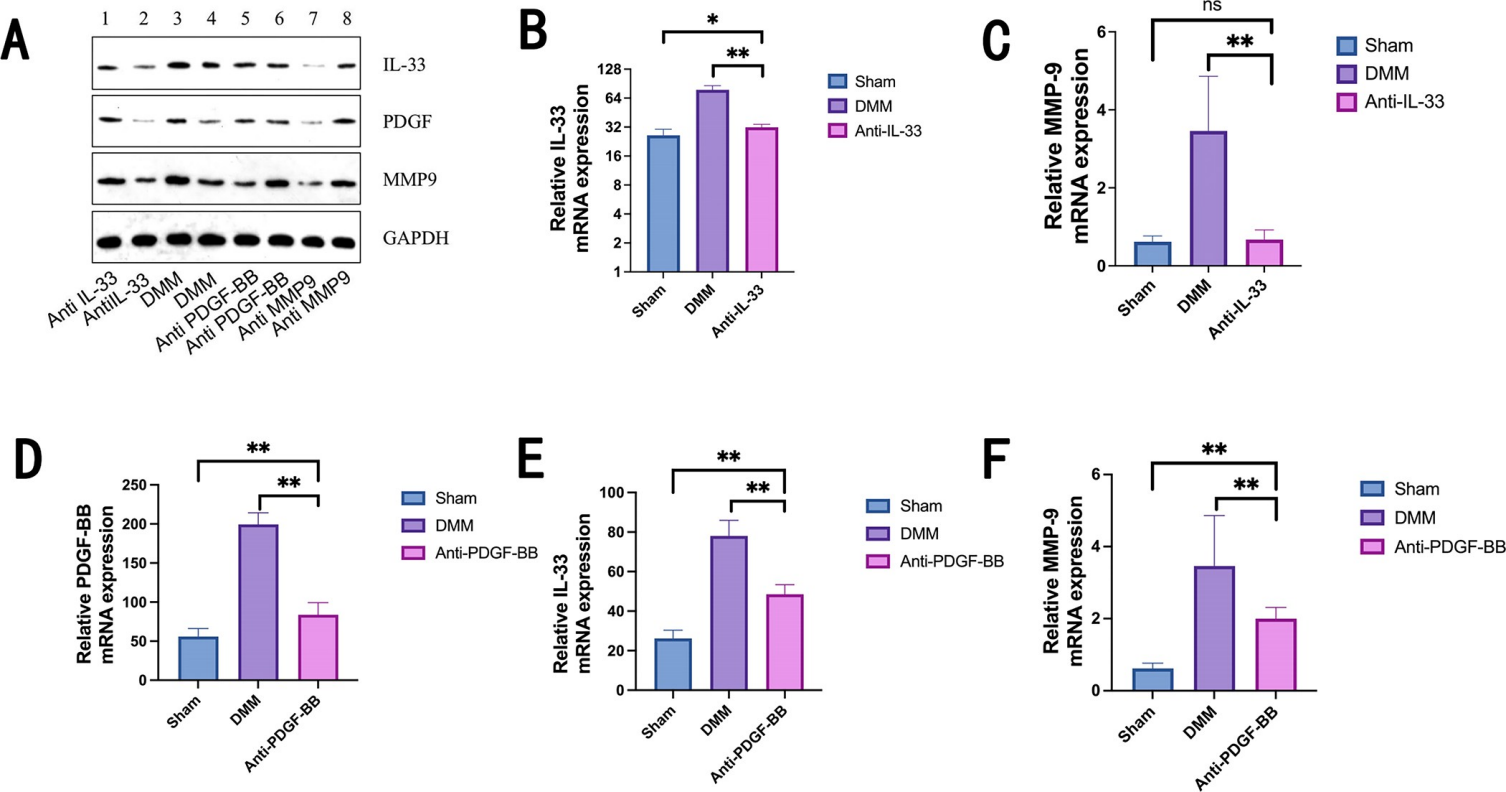
Effectiveness of antibody therapy in reducing inflammatory factors in murine knee cartilage: IL-33, PDGF-BB, and MMP-9 analysis. The expression of inflammatory factors in the mouse cartilage was reduced after four weeks of antibody therapy. Analysis of IL-33 by Western blot in Murine KOA of cartilage(A). According to Real-time PCR analysis (Tables 3 and 4), the relative mRNA expression levels of IL-33 and MMP-9 in the IL-33 antibody group were considerably reduced (P<0.01) after IL-33 and PDGF-BB antibody injection in mice knee four weeks (B, C). The relative mRNA expression levels of PDGF-BB, IL-33, and MMP-9 were all significantly lower in the PDGF-BB antibody group when compared to the control group (P<0.01) and significantly lower compared to the control group (P<0.05) (D, E, F). The one-way analysis of variance (ANOVA) test was used to analyze the data, and the double test between any two groups was performed using LSD and the S-N-K method. NS means no significant difference (P>0.05), * means significant difference (P<0.05), ** means extremely significant difference (P<0.01).

Real-time PCR and western blot analysis were used to assess the relative levels of inflammatory factor expression in murine KOA. After four weeks of antibody treatment, a significant difference in protein expression was observed (Fig 4A). The study demonstrated that although the relative mRNA expression levels for PDGF-BB, IL-33, and MMP-9 in the anti-PDGF-BB group were significantly higher than those in the sham group (*P*<0.01), they remained lower than those in the DMM group (*P*<0.01, Table 3 and Fig 4D–4F). While MMP-9 did not show a statistically significant difference from the blank group (*P*>0.05), the relative mRNA expression levels of IL-33 and MMP-9 were significantly lower in the IL-33 antibody group compared to the model control group (*P*<0.01). IL-33 did not exhibit a significant difference compared to the blank group (*P*>0.05, Table 4 and Fig 4B and 4C).

**Table 3:** PDGF-BB antibody group vs. control group (Mean ± SD).

Grouping	Cases	PDGF-BB mRNA	IL-33 mRNA	MMP-9 mRNA
Anti-PDGF-BB	9	83.82220 ± 15.35365	48.55000 ± 4.80414	2.00000 ± 0.31293
DMM	9	199.53670 ± 14.65879 **	78.02220 ± 7.97621 **	3.46000 ± 1.39939 **
Sham	9	55.92440 ± 10.27673 **	26.24670 ± 4.10570 **	0.62000 ± 0.14756 **

**Table 4:** Relative expression levels in IL-33 antibody vs. control group (Mean ± SD).

Grouping	Cases	IL-33 mRNA	MMP-9 mRNA
Anti-IL 33	9	31.88890 ± 2.33789	0.67440 ± 0.24815
DMM	9	78.02220 ± 7.97621 **	3.46000 ± 1.39939 **
Sham	9	26.24670 ± 4.10570*	0.62000 ± 0.14756 ns

#### Distinct attenuation of KOA with IL-33 blockade

**Fig 5:**
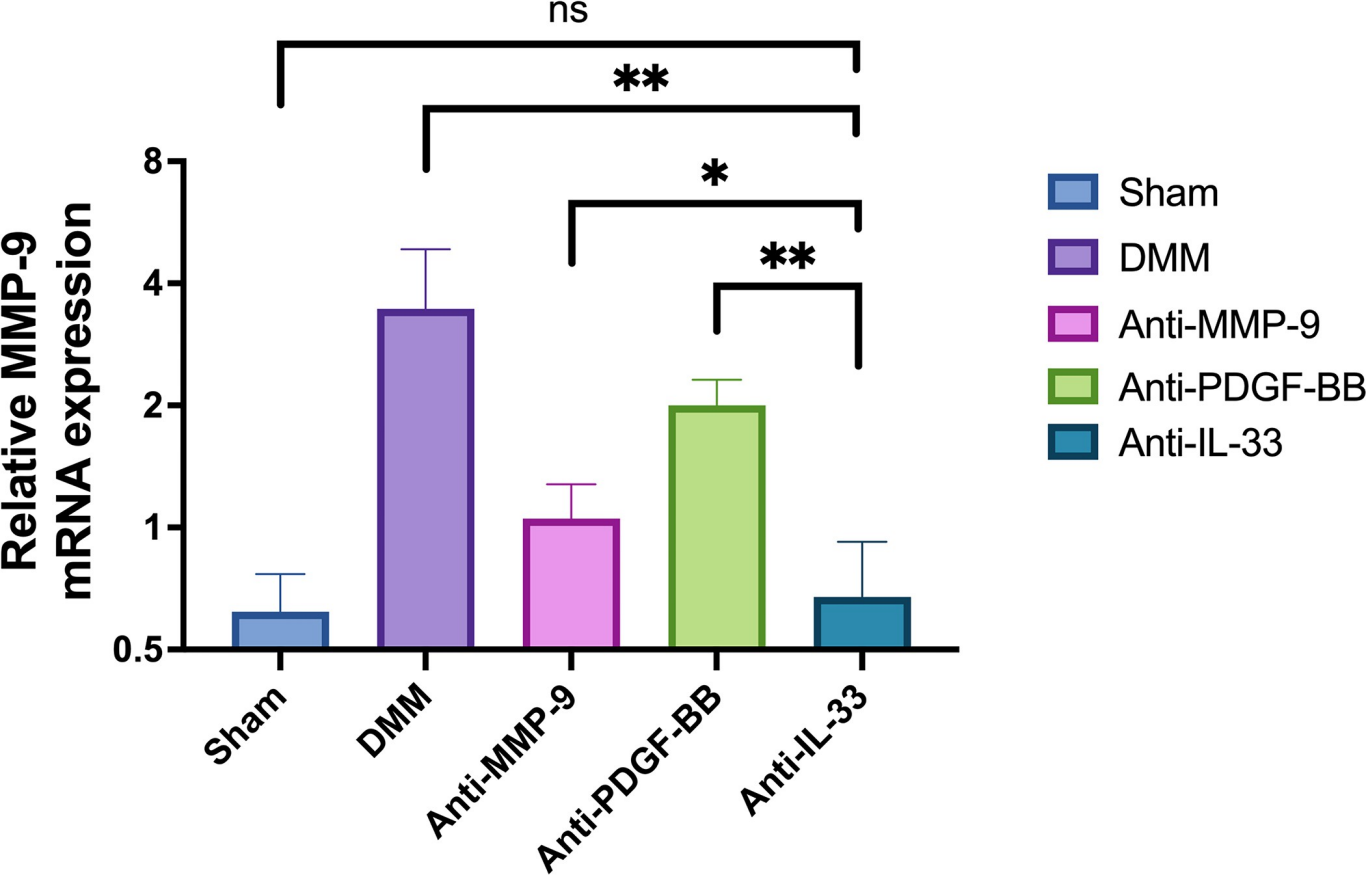
Differential suppression of MMP-9 expression by antibody therapy in murine knee cartilage: IL-33, PDGF-BB, and MMP-9 comparison. Comparison of relative expression levels of MMP-9. The relative MMP-9 expression levels in the MMP-9 antibody group, PDGF-BB antibody group, and IL-33 antibody group were lower than those in the control group (*P*<0.01). There was a substantial difference between the IL-33 antibody group and the MMP-9 antibody group, with the IL-33 antibody group having the lowest MMP-9 content (*P*<0.05). One-way analysis of variance (ANOVA) test and LSD and S-N-K double test were used to analyze the data between any two groups. Ns means no significant difference (*P*>0.05), *means significant difference (*P*<0.05), ** means extremely significant difference (*P*<0.01).

To assess the impact of IL-33 blockade on KOA, the relative mRNA expression levels of MMP-9 were measured using real-time PCR (Table 5 and Fig 5). The results demonstrate that the relative expression level of MMP-9 could be reduced by PDGF-BB antibody, IL-33 antibody, and MMP-9 antibody, with the PDGF-BB antibody group exhibiting the highest reduction, and the IL-33 antibody group showing the least reduction. The IL-33 antibody group and MMP-9 antibody group exhibited a significant difference from the PDGF-BB antibody group (P<0.01). Additionally, a significant difference existed between the IL-33 antibody group and the MMP-9 antibody group (P<0.05), while no significant difference was observed between the IL-33 antibody group and the control group (P>0.05).

**Table 5:** Comparison of relative expression mRNA levels of MMP-9 (MEAN±SD).

Grouping	Cases	MMP-9 mRNA
Sham	9	0.62000 ± 0.14756
DMM	9	3.46000 ± 1.39939 **
Anti-MMP-9	9	1.05220 ± 0.22565*
Anti-PDGF-BB	9	2.00000 ± 0.31293 **
Anti-IL-33	9	0.67440 ± 0.24815 ns

### Discuss and analysis

Our research establishes strong correlations between PDGF-BB, IL-33, MMP-9, and KOA. We conducted interventions with PDGF-BB and IL-33 antibodies in KOA model mice to explore the relationships among these factors. Importantly, we found that PDGF-BB inhibition led to a reduction in the relative expression levels of MMP-9 and IL-33. Furthermore, we identified PDGF-BB as an upstream factor of IL-33, and MMP-9 as a downstream factor of IL-33 in the KOA progression pathway. These findings highlight the need for further investigations into the interactions between these pathways.

Additionally, a study by Lianfu Deng and colleagues [23] corroborates our findings by revealing that the TLR3-p38-MAPK-NF-κB pathway can induce chondrocytes to secrete substantial IL-33, and the IL-33-ST2 pathway can trigger the production of proteases like MMP-9 and MMP-13, accelerating cartilage degradation. Our results demonstrated that, when comparing the efficacy in inhibiting MMP-9, the IL-33 antibody group exhibited the lowest efficacy compared to the control group, followed by the MMP-9 antibody group and the PDGF-BB antibody group. This suggests that the IL-33 antibody has a more pronounced advantage in suppressing MMP-9.

Following 4 weeks of treatment post-modeling, in combination with the pathological findings, the IL-33 antibody displayed substantial efficacy in preventing KOA progression and slowing down cartilage degradation.

Interleukin 33 (IL-33), also known as IL-1F11, is a member of the IL-1 cytokine family [31,32] and exerts its effects by binding to a heterodimeric receptor complex consisting of Suppression of Tumorigenicity2 (ST2) and IL-1 receptor accessory proteins [33,34]. IL-33 is released by endothelial and epithelial cells in response to stress, serving as a trigger for inflammatory responses [16,35]. It concurrently influences macrophage polarization and dendritic cell modulation, primarily inducing inflammatory responses and fibrosis. Research has demonstrated the link between IL-33 expression and the etiology of KOA, indicating that KOA can lead to elevated IL-33 levels [22,36]. Our study identified higher levels of IL-33 expression in DMM mice compared to the control group, further solidifying the strong association between IL-33 and KOA. Additionally, the DMM mouse model exhibited significant expression of PDGF-BB and MMP-9.

Recent research has emphasized that fibrosis, in addition to inflammation, is a key pathogenic mechanism in KOA. PDGF-BB and PDGFR-β interactions in fibroblasts have been extensively studied in diseases like rheumatoid arthritis [37,38], although not in KOA. Macrophages, fibroblasts, and endothelial cells can produce and release PDGF in response to tissue damage, with PDGF-BB inducing angiogenesis in subchondral bone. In the early stages of KOA, there is a rapid increase in subchondral bone angiogenesis, and the invasion of blood vessels through the cartilage tide line into avascular cartilage is a distinctive feature of human osteoarthritis. Abnormal articular subchondral bone angiogenesis, believed to accelerate osteoarthritis progression, is established before articular cartilage injury following medial meniscus (DMM) instability in mice [39]. This process, involving the formation of new blood vessels from preexisting ones within the osteoarthritic joint, is thought to expedite osteoarthritis progression and is associated with various diseases, including cancer and tissue fibrosis. Subchondral mononuclear osteoclasts release excessive PDGF-BB, promoting the proliferation and migration of perivascular cells by activating PDGFR-β [26]. Previous research has indicated that IL-33 is the most up-regulated gene in PDGF-BB-stimulated pericytes and that the SOX7 transcription factor facilitates PDGF-BB-induced IL-33 expression [28]. Conversely, IL-33 can stimulate macrophages by binding to their ST2 receptors [40,41], leading to the secretion of MMP-9, MMP-13, and other inflammatory factors [11,42]. In a study on tumor disease, it was also found that IL-33 may activate the ST2-NF-κB signaling pathway, increasing the expression of MMP-2 and MMP-9 [43]. MMP-9 is part of the MMPs family and can be released by various cells, including synoviocytes, macrophages, and neutrophils [44,45]. According to studies [46], MMP-2 and MMP-9 levels in KOA patients were significantly higher than those of the other seven MMPs (MMP-1, MMP-2, MMP-3, MMP-7, MMP-8, MMP-9, and MMP-13). Our investigation precisely identified the expression of MMP-9, revealing significantly higher levels in KOA patients compared to healthy controls.

In our study, the pathological knee joint cartilage samples from the surgical model group indicated chondrocyte destruction, and real-time PCR results demonstrated a significant increase in PDGF-BB, IL-33, and MMP-9, affirming the strong connection between these three proteins and knee osteoarthritis. However, after antibody blocking treatment, it was evident that the IL-33 antibody had a significantly greater inhibitory effect than the MMP-9 antibody, suggesting that IL-33 operates upstream in the MMP-9 pathway, influencing its expression during KOA development. This implies that a potential MMP-9 production pathway can be targeted to reduce downstream MMP-9 synthesis, subsequently mitigating extracellular matrix degradation in cartilage, as well as chondrocyte and subchondral bone degeneration. Our experimental findings indicated that, in terms of the degree of knee cartilage damage, the DMM group displayed the most severe damage, followed by the model control group. After 4 weeks of IL-33 antibody injections, the knee cartilage structure for mice with KOA demonstrated significantly better integrity compared to the model control group, the MMP-9 antibody group, and the PDGF-BB antibody group. These results illustrated the substantial inhibitory effect of the IL-33 antibody in preventing cartilage breakdown and degradation.

While PDGF-BB is frequently used in clinical KOA treatments with PRP agents, it is associated with abnormal angiogenesis in the early stages of the disease. Therefore, the application of PDGF-BB antibody in KOA mice may impact knee cartilage healing.

The findings of our study hold promise for the development of targeted therapies for KOA, shedding light on the potential of IL-33 and its interactions with other factors in the disease progression pathway. Further research is warranted to explore the intricate interplay among PDGF-BB, IL-33, and MMP-9, and to translate these discoveries into clinical applications for the benefit of KOA patients.

In our study, it was inevitable to elucidate some limitations regarding the experimental design. We solely employed the KOA DMM model. Using alternative models, such as the anterior cruciate ligament transection model, could further substantiate our findings. Additionally, histopathological examination revealed remarkable therapeutic effects of IL-33 antibody administration at the 4-week time point post-modeling, in terms of suppressing and slowing KOA progression and cartilage destruction. Therefore, IL-33 might serve as another promising therapeutic target for KOA pathogenesis. However, there may be time-dependent differences, as we only assessed the 4-week time point post-modeling in this study. According to current understanding of DMM modeling [29], mice are typically in the early to moderate stages of KOA currently point. Hence, further research is needed to investigate the relationship and comparative efficacy of these interventions in the mid to late stages of KOA.

## Conclusion

In conclusion, our research suggests that blocking IL-33 signaling could be a promising therapeutic approach for KOA. Furthermore, we suggest that PDGF-BB may serve as an upstream regulator of the IL-33 pathway, whereas MMP-9 seems to act as a downstream modulator of IL-33 in the context of KOA. These findings lay the groundwork for additional research into targeted therapies for KOA. The intricate interactions among these pivotal factors in the disease pathway offer potential for clinical applications in KOA management.

## Supporting information

S1 Raw images Original images: Original western blotting images in Fig 2. (PDF)

S2 Raw images Original images: Original western blotting images in Fig 4. (PDF)

## References

[1] Hunter DJ, Bierma-Zeinstra S. Osteoarthritis. Lancet. 2019;393(10182):1745–59. Epub 2019/04/30. doi: 10.1016/S0140-6736(19)30417-9 . 31034380

[2] Global, regional, and national incidence, prevalence, and years lived with disability for 354 diseases and injuries for 195 countries and territories, 1990–2017: a systematic analysis for the Global Burden of Disease Study 2017. Lancet. 2018;392(10159):1789–858. Epub 2018/11/30. doi: 10.1016/S0140-6736(18)32279-7 ; PubMed Central PMCID: PMC6227754. 30496104 PMC6227754

[3] Palazzo C, Nguyen C, Lefevre-Colau MM, Rannou F, Poiraudeau S. Risk factors and burden of osteoarthritis. Ann Phys Rehabil Med. 2016;59(3):134–8. Epub 2016/02/26. doi: 10.1016/j.rehab.2016.01.006 . 26904959

[4] Litwic A, Edwards MH, Dennison EM, Cooper C. Epidemiology and burden of osteoarthritis. Br Med Bull. 2013;105:185–99. Epub 2013/01/23. doi: 10.1093/bmb/lds038 ; PubMed Central PMCID: PMC3690438. 23337796 PMC3690438

[5] Chen D, Shen J, Zhao W, Wang T, Han L, Hamilton JL, et al. Osteoarthritis: toward a comprehensive understanding of pathological mechanism. Bone Res. 2017;5:16044. Epub 2017/02/06. doi: 10.1038/boneres.2016.44 ; PubMed Central PMCID: PMC5240031. 28149655 PMC5240031

[6] Zeng N, Yan ZP, Chen XY, Ni GX. Infrapatellar Fat Pad and Knee Osteoarthritis. Aging Dis. 2020;11(5):1317–28. Epub 2020/10/06. doi: 10.14336/AD.2019.1116 ; PubMed Central PMCID: PMC7505265. 33014539 PMC7505265

[7] Donell S. Subchondral bone remodelling in osteoarthritis. EFORT Open Rev. 2019;4(6):221–9. Epub 2019/06/19. doi: 10.1302/2058-5241.4.180102 ; PubMed Central PMCID: PMC6549114. 31210964 PMC6549114

[8] Belluzzi E, Stocco E, Pozzuoli A, Granzotto M, Porzionato A, Vettor R, et al. Contribution of Infrapatellar Fat Pad and Synovial Membrane to Knee Osteoarthritis Pain. Biomed Res Int. 2019;2019:6390182. Epub 2019/05/03. doi: 10.1155/2019/6390182 ; PubMed Central PMCID: PMC6462341. 31049352 PMC6462341

[9] Malemud CJ. Biologic basis of osteoarthritis: state of the evidence. Curr Opin Rheumatol. 2015;27(3):289–94. Epub 2015/03/19. doi: 10.1097/BOR.0000000000000162 ; PubMed Central PMCID: PMC4492522. 25784380 PMC4492522

[10] Pelletier JP, Martel-Pelletier J, Abramson SB. Osteoarthritis, an inflammatory disease: potential implication for the selection of new therapeutic targets. Arthritis Rheum. 2001;44(6):1237–47. Epub 2001/06/16. doi: 10.1002/1529-0131(200106)44:6&lt;1237::AID-ART214&gt;3.0.CO;2-F . 11407681

[11] Wynn TA, Vannella KM. Macrophages in Tissue Repair, Regeneration, and Fibrosis. Immunity. 2016;44(3):450–62. Epub 2016/03/18. doi: 10.1016/j.immuni.2016.02.015 ; PubMed Central PMCID: PMC4794754. 26982353 PMC4794754

[12] Andreas N, Weber F, Meininger I, Templin N, Gaestel M, Kamradt T, et al. IL-33-activated murine mast cells control the dichotomy between RORγt(+) and Helios(+) T(regs) via the MK2/3-mediated IL-6 production in vitro. Eur J Immunol. 2019;49(12):2159–71. Epub 2019/07/25. doi: 10.1002/eji.201948154 . 31334837

[13] Hong J, Kim S, Lin PC. Interleukin-33 and ST2 Signaling in Tumor Microenvironment. J Interferon Cytokine Res. 2019;39(1):61–71. Epub 2018/09/27. doi: 10.1089/jir.2018.0044 ; PubMed Central PMCID: PMC6350413. 30256696 PMC6350413

[14] Weber AE, Bolia IK, Trasolini NA. Biological strategies for osteoarthritis: from early diagnosis to treatment. Int Orthop. 2021;45(2):335–44. Epub 2020/10/21. doi: 10.1007/s00264-020-04838-w . 33078204

[15] Ruan G, Xu J, Wang K, Zheng S, Wu J, Bian F, et al. Associations between serum IL-8 and knee symptoms, joint structures, and cartilage or bone biomarkers in patients with knee osteoarthritis. Clin Rheumatol. 2019;38(12):3609–17. Epub 2019/08/05. doi: 10.1007/s10067-019-04718-8 . 31377918

[16] Liew FY, Girard JP, Turnquist HR. Interleukin-33 in health and disease. Nat Rev Immunol. 2016;16(11):676–89. Epub 2016/10/27. doi: 10.1038/nri.2016.95 . 27640624

[17] Saikumar Jayalatha AK, Hesse L, Ketelaar ME, Koppelman GH, Nawijn MC. The central role of IL-33/IL-1RL1 pathway in asthma: From pathogenesis to intervention. Pharmacol Ther. 2021;225:107847. Epub 2021/04/06. doi: 10.1016/j.pharmthera.2021.107847 . 33819560

[18] Hodzic Z, Schill EM, Bolock AM, Good M. IL-33 and the intestine: The good, the bad, and the inflammatory. Cytokine. 2017;100:1–10. Epub 2017/07/09. doi: 10.1016/j.cyto.2017.06.017 ; PubMed Central PMCID: PMC5650929. 28687373 PMC5650929

[19] Cayrol C, Girard JP. Interleukin-33 (IL-33): A critical review of its biology and the mechanisms involved in its release as a potent extracellular cytokine. Cytokine. 2022;156:155891. Epub 2022/06/01. doi: 10.1016/j.cyto.2022.155891 35640416

[20] Mehana EE, Khafaga AF, El-Blehi SS. The role of matrix metalloproteinases in osteoarthritis pathogenesis: An updated review. Life Sci. 2019;234:116786. Epub 2019/08/26. doi: 10.1016/j.lfs.2019.116786 . 31445934

[21] Rai V, Radwan MM, Agrawal DK. IL-33, IL-37, and Vitamin D Interaction Mediate Immunomodulation of Inflammation in Degenerating Cartilage. Antibodies (Basel). 2021;10(4). Epub 2021/11/30. doi: 10.3390/antib10040041 ; PubMed Central PMCID: PMC8628513. 34842603 PMC8628513

[22] Rai V, Dilisio MF, Samadi F, Agrawal DK. Counteractive Effects of IL-33 and IL-37 on Inflammation in Osteoarthritis. Int J Environ Res Public Health. 2022;19(9). Epub 2022/05/15. doi: 10.3390/ijerph19095690 ; PubMed Central PMCID: PMC9100324. 35565085 PMC9100324

[23] Li C, Chen K, Kang H, Yan Y, Liu K, Guo C, et al. Double-stranded RNA released from damaged articular chondrocytes promotes cartilage degeneration via Toll-like receptor 3-interleukin-33 pathway. Cell Death Dis. 2017;8(11):e3165. Epub 2017/11/03. doi: 10.1038/cddis.2017.534 ; PubMed Central PMCID: PMC5775407. 29095435 PMC5775407

[24] Walsh DA, McWilliams DF, Turley MJ, Dixon MR, Fransès RE, Mapp PI, et al. Angiogenesis and nerve growth factor at the osteochondral junction in rheumatoid arthritis and osteoarthritis. Rheumatology (Oxford). 2010;49(10):1852–61. Epub 2010/06/29. doi: 10.1093/rheumatology/keq188 ; PubMed Central PMCID: PMC2936950. 20581375 PMC2936950

[25] Mapp PI, Walsh DA. Mechanisms and targets of angiogenesis and nerve growth in osteoarthritis. Nat Rev Rheumatol. 2012;8(7):390–8. Epub 2012/05/30. doi: 10.1038/nrrheum.2012.80 . 22641138

[26] Su W, Liu G, Liu X, Zhou Y, Sun Q, Zhen G, et al. Angiogenesis stimulated by elevated PDGF-BB in subchondral bone contributes to osteoarthritis development. JCI Insight. 2020;5(8). Epub 2020/03/26. doi: 10.1172/jci.insight.135446 ; PubMed Central PMCID: PMC7205438. 32208385 PMC7205438

[27] Hu Y, Chen X, Wang S, Jing Y, Su J. Subchondral bone microenvironment in osteoarthritis and pain. Bone Res. 2021;9(1):20. Epub 2021/03/19. doi: 10.1038/s41413-021-00147-z ; PubMed Central PMCID: PMC7969608. 33731688 PMC7969608

[28] Yang Y, Andersson P, Hosaka K, Zhang Y, Cao R, Iwamoto H, et al. The PDGF-BB-SOX7 axis-modulated IL-33 in pericytes and stromal cells promotes metastasis through tumour-associated macrophages. Nat Commun. 2016;7:11385. Epub 2016/05/07. doi: 10.1038/ncomms11385 ; PubMed Central PMCID: PMC4859070. 27150562 PMC4859070

[29] Glasson SS, Blanchet TJ, Morris EA. The surgical destabilization of the medial meniscus (DMM) model of osteoarthritis in the 129/SvEv mouse. Osteoarthritis Cartilage. 2007;15(9):1061–9. Epub 2007/05/02. doi: 10.1016/j.joca.2007.03.006 . 17470400

[30] Lorenz J, Grässel S. Experimental osteoarthritis models in mice. Methods Mol Biol. 2014;1194:401–19. Epub 2014/07/30. doi: 10.1007/978-1-4939-1215-5_23 . 25064117

[31] Haraldsen G, Balogh J, Pollheimer J, Sponheim J, Küchler AM. Interleukin-33—cytokine of dual function or novel alarmin? Trends Immunol. 2009;30(5):227–33. Epub 2009/04/11. doi: 10.1016/j.it.2009.03.003 . 19359217

[32] Kakkar R, Lee RT. The IL-33/ST2 pathway: therapeutic target and novel biomarker. Nat Rev Drug Discov. 2008;7(10):827–40. Epub 2008/10/02. doi: 10.1038/nrd2660 ; PubMed Central PMCID: PMC4277436. 18827826 PMC4277436

[33] Griesenauer B, Paczesny S. The ST2/IL-33 Axis in Immune Cells during Inflammatory Diseases. Front Immunol. 2017;8:475. Epub 2017/05/10. doi: 10.3389/fimmu.2017.00475 ; PubMed Central PMCID: PMC5402045. 28484466 PMC5402045

[34] Jiang W, Lian J, Yue Y, Zhang Y. IL-33/ST2 as a potential target for tumor immunotherapy. Eur J Immunol. 2021;51(8):1943–55. Epub 2021/06/17. doi: 10.1002/eji.202149175 . 34131922

[35] Cayrol C, Girard JP. Interleukin-33 (IL-33): A nuclear cytokine from the IL-1 family. Immunol Rev. 2018;281(1):154–68. Epub 2017/12/17. doi: 10.1111/imr.12619 . 29247993

[36] He Z, Song Y, Yi Y, Qiu F, Wang J, Li J, et al. Blockade of IL-33 signalling attenuates osteoarthritis. Clin Transl Immunology. 2020;9(10):e1185. Epub 2020/11/03. doi: 10.1002/cti2.1187 ; PubMed Central PMCID: PMC7587452. 33133598 PMC7587452

[37] Cao Y. Multifarious functions of PDGFs and PDGFRs in tumor growth and metastasis. Trends Mol Med. 2013;19(8):460–73. Epub 2013/06/19. doi: 10.1016/j.molmed.2013.05.002 . 23773831

[38] Andrae J, Gallini R, Betsholtz C. Role of platelet-derived growth factors in physiology and medicine. Genes Dev. 2008;22(10):1276–312. Epub 2008/05/17. doi: 10.1101/gad.1653708 ; PubMed Central PMCID: PMC2732412. 18483217 PMC2732412

[39] Li B, Chen K, Qian N, Huang P, Hu F, Ding T, et al. Baicalein alleviates osteoarthritis by protecting subchondral bone, inhibiting angiogenesis and synovial proliferation. J Cell Mol Med. 2021;25(11):5283–94. Epub 2021/05/04. doi: 10.1111/jcmm.16538 ; PubMed Central PMCID: PMC8178278. 33939310 PMC8178278

[40] Xiong Z, Thangavel R, Kempuraj D, Yang E, Zaheer S, Zaheer A. Alzheimer’s disease: evidence for the expression of interleukin-33 and its receptor ST2 in the brain. J Alzheimers Dis. 2014;40(2):297–308. Epub 2014/01/15. doi: 10.3233/JAD-132081 ; PubMed Central PMCID: PMC4015800. 24413615 PMC4015800

[41] Wang X, Yu YY, Lieu S, Yang F, Lang J, Lu C, et al. MMP9 regulates the cellular response to inflammation after skeletal injury. Bone. 2013;52(1):111–9. Epub 2012/09/27. doi: 10.1016/j.bone.2012.09.018 ; PubMed Central PMCID: PMC3513654. 23010105 PMC3513654

[42] Ji B, Ma Y, Wang H, Fang X, Shi P. Activation of the P38/CREB/MMP13 axis is associated with osteoarthritis. Drug Des Devel Ther. 2019;13:2195–204. Epub 2019/07/17. doi: 10.2147/DDDT.S209626 ; PubMed Central PMCID: PMC6613348. 31308631 PMC6613348

[43] Zhang JF, Wang P, Yan YJ, Li Y, Guan MW, Yu JJ, et al. IL‑33 enhances glioma cell migration and invasion by upregulation of MMP2 and MMP9 via the ST2-NF-κB pathway. Oncol Rep. 2017;38(4):2033–42. Epub 2017/08/30. doi: 10.3892/or.2017.5926 ; PubMed Central PMCID: PMC5652951. 28849217 PMC5652951

[44] Vandooren J, Van den Steen PE, Opdenakker G. Biochemistry and molecular biology of gelatinase B or matrix metalloproteinase-9 (MMP-9): the next decade. Crit Rev Biochem Mol Biol. 2013;48(3):222–72. Epub 2013/04/04. doi: 10.3109/10409238.2013.770819 . 23547785

[45] Burrage PS, Mix KS, Brinckerhoff CE. Matrix metalloproteinases: role in arthritis. Front Biosci. 2006;11:529–43. Epub 2005/09/09. doi: 10.2741/1817 . 16146751

[46] Tetlow LC, Adlam DJ, Woolley DE. Matrix metalloproteinase and proinflammatory cytokine production by chondrocytes of human osteoarthritic cartilage: associations with degenerative changes. Arthritis Rheum. 2001;44(3):585–94. Epub 2001/03/27. doi: 10.1002/1529-0131(200103)44:3&lt;585::AID-ANR107&gt;3.0.CO;2-C . 11263773

