# Supplementary figures and images for: Blocking IL-33 decelerates cartilage degeneration in knee osteoarthritis through mice model
Source: PLoS One. 2024 Aug 22;19(8):e0301199. doi: 10.1371/journal.pone.0301199 (PMC11340949; doi:10.1371/journal.pone.0301199)

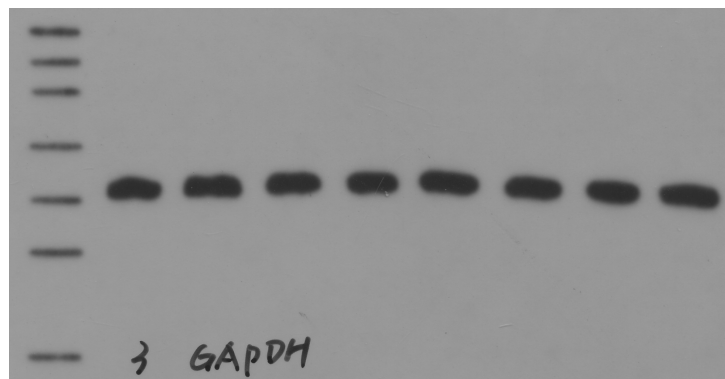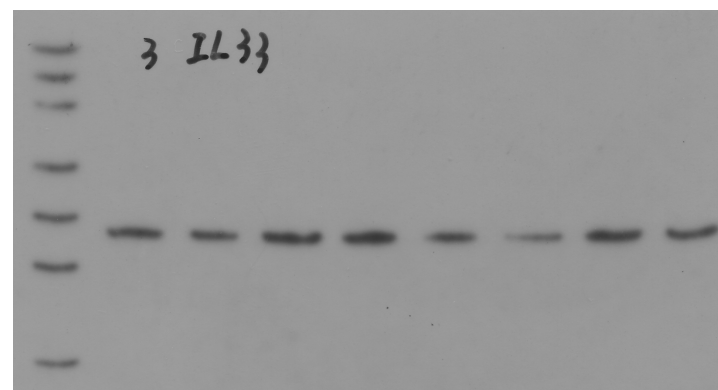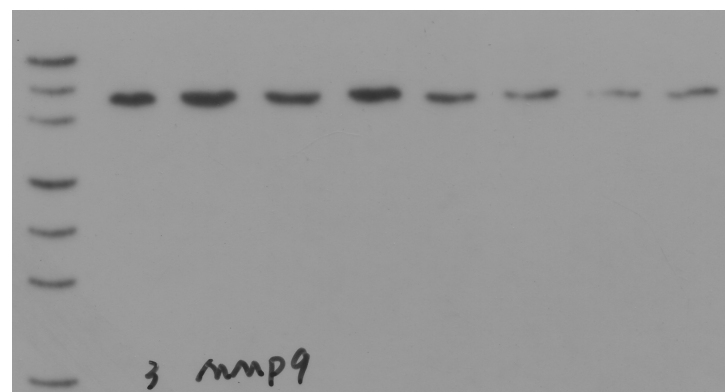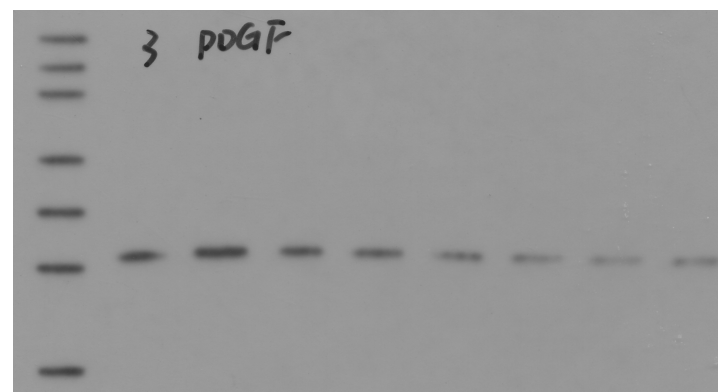

Supplement: S1 Raw images — (PDF) [file pone.0301199.s001.pdf]

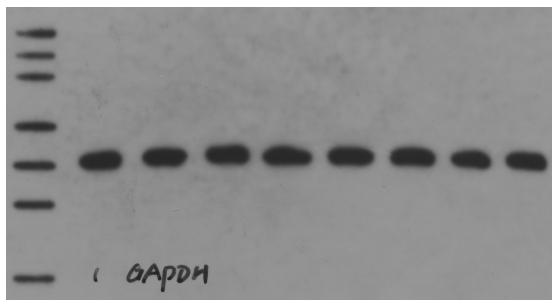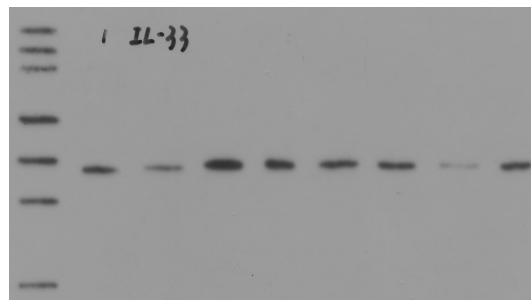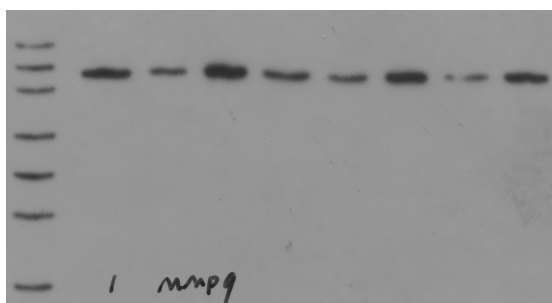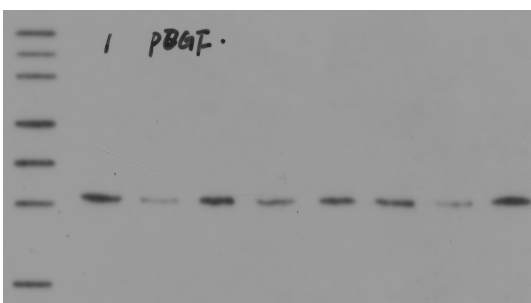

Supplement: S2 Raw images — (PDF) [file pone.0301199.s002.pdf]
